# Characterisation of the *GRAF* gene promoter and its methylation in patients with acute myeloid leukaemia and myelodysplastic syndrome

**DOI:** 10.1038/sj.bjc.6602939

**Published:** 2006-01-10

**Authors:** S E Bojesen, O Ammerpohl, A Weinhäusl, O A Haas, H Mettal, R M Bohle, A Borkhardt, U Fuchs

**Affiliations:** 1 Department of Clinical Biochemistry, Copenhagen University Hospital, Herlev, Denmark; 2 University Hospital of Schleswig-Holstein, Clinic for General Surgery and Thoracic Surgery, Kiel, Germany; 3 ARCS, Austrian Research Center, Seibersdorf, Austria; 4 CCRI, Vienna, Austria; 5 University Hospital of Tübingen, Clinic for Anaesthesiology and transfusion medicine, Tübingen, Germany; 6 University Hospital of Giessen, Institute of Pathology, Giessen, Germany; 7 Dr von Haunersches Kinderspital, Department of Haematology/Oncology, Munich, Germany

**Keywords:** *GRAF*, AML, promoter methylation, MDS

## Abstract

We report the isolation of the 5′ flanking region of *GRAF* (*G*TPase *r*egulator *a*ssociated with the *f*ocal adhesion kinase), previously described as a putative tumour suppressor gene of acute myelogenous leukaemia and myelodysplastic syndrome, and demonstrate its promoter activity in reporter gene assays. Two putative protein-binding sites are identified of which one was sensitive to CpG methylation. The suppressed *GRAF* expression could be restored in leukaemia cell lines by treatment with a demethylating agent and an inhibitor of histone deacetylases. In contrast to normal tissues, which tested negative for *GRAF* promoter methylation, 11 of 29 (38%) bone marrow samples from patients with acute myeloid leukaemia or myelodysplastic syndrome were positive.

The *GRAF* (*G*TPase *r*egulator *a*ssociated with the *f*ocal adhesion kinase) gene at chromosome 5q31 has been identified as a translocation partner of *MLL* (also known as *ALL-1*, *HRX* or *HTRX*) in a case of juvenile myelomonocytic leukaemia (Borkhardt *et al*, 2000). GRAF has some unusual features with regard to its tumour suppressor properties. It is localised in the cytoplasm and negatively regulates the small GTP-binding protein RhoA (Hildebrand *et al*, 1996), which in turn is well known for its growth-promoting effect in RAS-mediated malignant transformation (Qiu *et al*, 1995).

*GRAF* function is affected by mutations and deletions of the gene in patients with acute myeloid leukaemia (AML) or myelodysplastic syndrome (MDS) (Borkhardt *et al*, 2000) and we hypothesised that *GRAF* function could also be affected by downregulation induced by methylation of its promoting regions as shown for established tumour suppressor genes (e.g. *hMLH1, HIC1, E-cad, VHL, CDKN2, RB, BRCA1*) (Herman *et al*, 1994, 1998; Gonzalez-Zulueta *et al*, 1995; Yoshiura *et al*, 1995; Dobrovic and Simpfendorfer, 1997; Graff *et al*, 1997; Jarrard *et al*, 1997; Ohtani-Fujita *et al*, 1997; Melki *et al*, 1999). Thus, we isolated the 5′ regulatory region of *GRAF* by genome walking, characterised it by reporter gene assays and looked therein for methylation-sensitive protein-binding sites. We tested partially and fully methylated alleles of the *GRAF* promoter by luciferase reporter gene assays. The influence of epigenetic modifications on the *GRAF* expression was further studied by quantitative PCR in leukaemia and lymphoma cell lines of different origin after treatment with the demethylating agent 5-aza-2′-deoxycytidine (5-azadC) or the histone deacetylase inhibitor trichostatin A (TSA).

Finally, we screened a series of bone marrow samples from patients with AML or MDS by methylation-sensitive PCR (MS-PCR).

## MATERIALS AND METHODS

### 5′ rapid amplification of cloned ends

PCR products generated by a nested 5′ rapid amplification of cloned ends (RACE) with gene-specific primers (Table 1) against adaptor primers on an adaptor-ligated cDNA library from the cell line K562 (Clontech, Palo Alto, CA, USA) according to the manufacturer's specifications were cloned into pBluescriptIISK+ (Stratagene, Heidelberg, Germany). The five longest of 25 clones from three independent PCR experiments were sequenced (ABI/Perkin-Elmer, Langen, Germany).

### Genome walking and construction of reporter plasmids

The genome walking was performed as a nested hot-start long-range PCR (conditions available upon request) with the five human templates from the Genome Walker kit #K1803-1 (Clontech, Palo Alto, CA, USA). PCR products were cloned and sequenced. The sequence of the 5′ region thus obtained was used to generate PCR fragments, which were cloned into the pGL3-Basic vector using the restriction sites *Hind*III and *Bgl*II. Plasmids were sequenced before proceeding to the dual luciferase assay.

### Cell cultivation and transfection

The human chronic myeloid leukaemia cell line K562 was cultured in RPMI 1640 medium (Gibco BRL, Karlsruhe, Germany) supplemented with 10% fetal calf serum, 260 mg l^−1^ L-glutamine, 50 000 U l^−1^ penicillin and 50 mg l^−1^ streptomycin. The cells were split 1 : 10 once a week and split 1 : 10 4 days before each transfection experiment.

RAW264 is a murine macrophage cell line and was cultured in DMEM (Gibco BRL, Karlsruhe, Germany) supplemented with 10% fetal calf serum, 260 mg l^−1^ L-glutamine, 50 000 U l^−1^ penicillin and 50 mg l^−1^ streptomycin.

Daudi was established from a Burkitt's lymphoma. Jurkat is a human T-cell leukaemia cell line. Kasumi-1 was derived from an AML (M2) harbouring a t(8;21). Mutz-1 was established from a patient with MDS and Fanconi anaemia. These cell lines were cultivated under the same conditions as K562.

Trichostatin A was dissolved in ethanol and added to the cell medium (RPMI or DMEM) to an end concentration of 100–300 ng ml^−1^.

### Reporter gene assays

**Figure 1:**
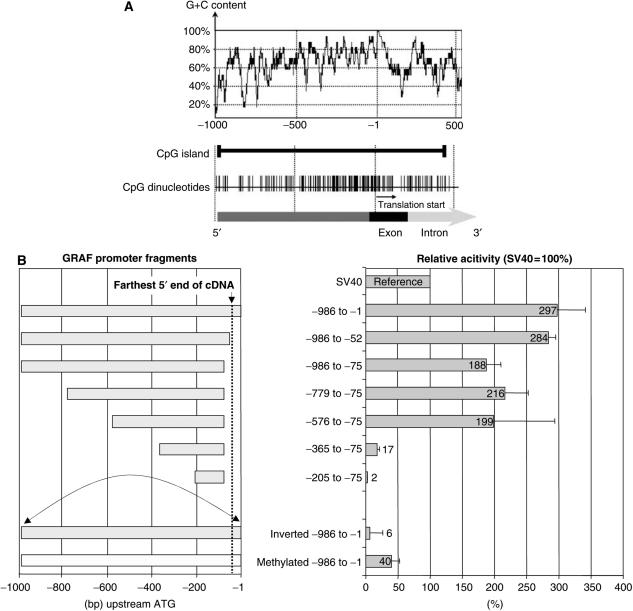
Overview (**A**) and deletion analyses (**B**) of the *GRAF* promoter. (**A**) The *GRAF* promoter is a GC-rich area with a dense concentration of CpG dinucleotides and a CpG island extending from −981 in the promoter to +350 in the downstream intron (numbering of nucleotides relative to A (=+1) in the START-ATG codon). Segment −986 to −1 was examined with reporter gene assays. (**B**) Left panel: The promoter segments (numbering relative to A (=+1) in the START-ATG codon). Right panel: Relative firefly luciferase activity normalised against the promoting activity of SV40 (=100%). K562 cells were transiently transfected with a pGL3 plasmid containing different segments of the *GRAF* promoter. Inversion (arrow) or methylation of the promoter completely abolishes any promoting activity. The data represent the mean±s.d. of three independent experiments.

K562 cells were transiently transfected with DMRIE-C (Gibco BRL, Karlsruhe, Germany). Reporter gene assays were performed using the dual luciferase reporter assay system (Promega, Mannheim, Germany) as described by the manufacturer. In short, the cells were co-transfected with two different plasmids, each coding for a luciferase: the *Renilla* luciferase coded for by the pRL-TK plasmid and the firefly luciferase coded for by the pGL3 plasmid. The two enzymes each have high specificity to their own substrates with minimal low cross-activities. For each transfection experiment, the activity of each enzyme was measured sequentially in a luminometer (AutoLumat LB953, Berthold, Bad Wildbad, Germany). The pRL-TK plasmid served as an internal control for each transfection, correcting for varying transfection efficiency. The relative activity of the pGL3 plasmid containing various fragments of the *GRAF* promoter was normalised against the activity of the SV40 promoter (=100%; Figure 1B) or the activity of the empty pGL3 plasmid (=1; Figure 3D and E).

### *In vitro* modifications of the *GRAF* promoter

A 40 *μ*g portion of the pGL3-Basic plasmid containing the –986 to −1 fragment of the *GRAF* promoter was digested with *Bgl*II and *Hind*III and separated by electrophoresis. The promoter fragment was excised from the agarose gel, purified and resolved. The fragment was methylated by M.*Sss*I methylase (New England Biolabs, Schwalbach, Germany) with 10 U enzyme *μ*g^−1^ DNA in the presence of 320 *μ*M *S*-adenosylmethionine (New England Biolabs, Schwalbach, Germany) for 60 min at 37°C. The methylation was controlled by digestion with the methylation-sensitive restriction enzyme *Bst*UI. The methylated DNA fragment and an unmethylated control fragment were each ligated into the pGL3-Basic vector prepared by *Bgl*II/*Hind*III digestion. After ligation had been confirmed by agarose gel analysis, the ligation mixture was purified using the Plasmid Mini Kit (Qiagen, Hilden, Germany). A 1 *μ*g portion was subsequently used for the dual luciferase assay (Figure 1B) as described above.

For the DNase I protection assays, the methyltransferases M.*Sss*I and M.*FnuD*II (New England Biolabs, Schwalbach, Germany) were used to methylate the promoter fragments according to the manufacturer's protocol.

The plasmid constructs with deleted putative DNA–protein-binding sites (Figure 3D) were generated by site-directed PCR mutagenesis according to Weiner *et al* (1994).

### Preparation of nuclear extracts and DNase I protection assays

Nuclear protein extracts from RAW264 and K562 cells were prepared as described previously (Dignam *et al*, 1983; Shapiro *et al*, 1988). Briefly, after nearly reaching confluence, cells from 100 cell culture dishes (15 cm) were harvested and resuspended in hypotonic buffer (for buffer compositions, see Supplementary Table C). Nuclei were released and sucrose restore buffer was added. After pelleting and lysing the nuclei by adding nuclear resuspension buffer, debris was removed by centrifugation and nuclear proteins were precipitated. Finally, the nuclear extract was dialysed against buffer D, aliquoted and stored at −80°C until use.

The unmethylated or methylated (by M.*FnuD*II or M.*Sss*I) promoter fragment (−576 to −75) was released from 10 *μ*g plasmid DNA by a *Bgl*II and *Hind*III digest and radioactively end-labelled with 15 U Klenow fragment in the presence of 50 *μ*Ci [*α*-^32^P]dGTP and non-radioactive dATP, dCTP and dTTP. The promoter fragment was isolated by agarose gel electrophoresis and 30 000 c.p.m. of DNA were incubated in binding buffer (Nickel *et al*, 1995) supplemented with 1 *μ*g of dIdC and various amounts of nuclear extracts as indicated in the figures. Subsequently, a DNase I digest was performed in a total volume of 60 *μ*l for 10 min on ice or at room temperature. The chosen incubation conditions only allowed an incomplete digestion, leaving a specific population of partially digested promoter fragments. The reaction was terminated by adding 2 volumes of stop buffer and 30 *μ*g of proteinase K and incubation at 55°C for 30 min. The promoter fragments were extracted and subjected to electrophoresis on a 6% polyacrylamide, 8 M urea gel, thereby separating the partially digested DNA fragments according to size. A G+A ladder served as a marker of size. Finally, the dried gel was autoradiographed on an X-ray film.

Nuclear proteins binding to the DNA could decrease the accessibility of the DNA for DNase I, thus leading to regions on the DNA protected from digestion. The binding could also induce conformational changes of the DNA, increasing the accessibility of the DNA for nucleases and leading to hypersensitive sites. Accordingly, DNase I protection/hypersensitivity is assumed to represent protein binding to the promoter fragment and is recognised as a change in the migration pattern from the lane without nuclear extract to the lanes with nuclear extract. In an attempt to quantify the effect, we used increasing amounts of DNase I or nuclear extract. If methylation of the promoter fragment is able to abolish this change of migration from the lane without nuclear extract to the lanes with nuclear extract, we interpret this as abolishment of protein binding.

### Quantification of *GRAF* mRNA by real-time PCR

For the quantification of the *GRAF* mRNA, a TaqMan™ PCR assay in combination with an ABI PRISM™ 7700 Sequence Detector (Applied Biosystems, Foster City, CA, USA) was used. After isolation of total RNA with Trizol™ reagent (Invitrogen, Karlsruhe, Germany), reverse transcription of 1 *μ*g of RNA was performed using Superscript II reverse transcriptase (Invitrogen, Karlsruhe, Germany) and oligo-dT primers (Sigma, Schnelldorf, Germany) according to the manufacturer's recommendations. A 1 *μ*l portion of this cDNA served as template in a 25 *μ*l two-step qPCR reaction (conditions are available upon request; for primer and probe sequences, see Table 1, PCR product: 81 bp). Each PCR was performed in triplicate and the number of *GRAF* mRNA molecules was determined according to a standard curve generated by a plasmid dilution series and normalised to the number of *ABL* mRNA molecules in a corresponding preparation.

### Patients’ samples

Bone marrow samples from 29 paediatric patients suffering from AML or MDS were collected in the framework of the Berlin–Frankfurt–Muenster (BFM) study group. The selection of cases was biased with regard to availability of frozen tumour material as a prerequisite for the analysis. All samples were sent by mail to the Oncogenetic Laboratory of the Children's University Hospital in Giessen, Germany. We were not aware of any differences in tissue processing among the 29 samples studied. The mononuclear cells were separated by Ficoll® density gradient centrifugation according to the manufacturer's instructions (Serva, Heidelberg, Germany). The cells were resuspended in 250 *μ*l PBS, shock frozen in liquid nitrogen and stored at −80°C until analyses.

### Methylation-sensitive PCR

The MS-PCR was performed basically as described by Herman *et al* (1996). In brief, 1 *μ*g genomic DNA in a volume of 70 *μ*l was denatured by NaOH treatment (final concentration 0.33 M) for 15 min at 37°C, 3 min at 95°C and 30 min at 80°C. Modification of unmethylated cytosine residues was performed by adding 1 ml of 3 M NaSO_3_ (Sigma, Schnelldorf, Germany) prewarmed at 55°C and incubation under mineral oil for 4 h in the dark at 55°C. Subsequently, the modified DNA was purified using the Geneclean Kit (Bio 101, Vista, CA, USA). DNA was resuspended in 20 *μ*l H_2_O and used immediately or stored at −20°C.

PCR was performed as a nested hot-start PCR (conditions available upon request) with the primers listed in Table 1.

In parallel, for each MS-PCR, appropriate PCR controls using unmethylated (negative control) and *in vitro*-methylated genomic DNA (positive control) isolated from a healthy volunteer were performed.

The MS-PCR products (126 bp) were visualised on a 2% agarose gel and sequenced.

## RESULTS

### Isolation and characterisation of the 5′ flanking region of *GRAF*

Multiple 5′ RACE-PCR products were sequenced and the 5′ farthest cDNA fragment began only 37 bp upstream of the first ATG codon (GenBank accession no. Y10388). Next, we isolated a sequence of 3080 bp upstream of the presumed translation initiation codon by genome walking (GenBank accession no. AF196313).

A CpG island/HTF (*Hpa*II tiny fragment) according to the definition of Gardiner-Garden and Frommer (1987) spanning the first 981 bp immediately upstream of the presumed translation initiation codon, an exon and the first 350 bp of the adjacent intron was identified (Figure 1A). We did not find a TATA box or other well-known transcription initiation signals.

Next, we performed reporter gene assays with a series of 5′- and 3′-deleted *GRAF* promoter constructs generated by inserting PCR products into the pGL3-Basic vector just upstream of the firefly luciferase gene. The intact 5′ flanking region of *GRAF* is a powerful promoter in K562 cells with about three times the activity of the SV40 promoter (Figure 1B). Deletion experiments demonstrated that the 5′ end of the region examined is of minor importance for the promoter activity. The smallest fragment with intact activity proved to be the −576 to −365 fragment (data not shown). Further 5′ deletions abolished the activity. The activity of the reversed segment −981 to −1 proved to be approximately 1/50 of that of the unreversed segment. Thus, the promoter activity of the 5′ flanking 981 bp is dependent upon direction. CpG methylation with the M.*Sss*I methylase diminished the activity of the −981 to −1 fragment to approximately 1/7 of the activity of the unmethylated construct, which suggests that binding of *trans*-acting factors might be dependent upon DNA demethylation. We therefore tried to identify these sites using DNase I protection assays.

**Figure 2:**
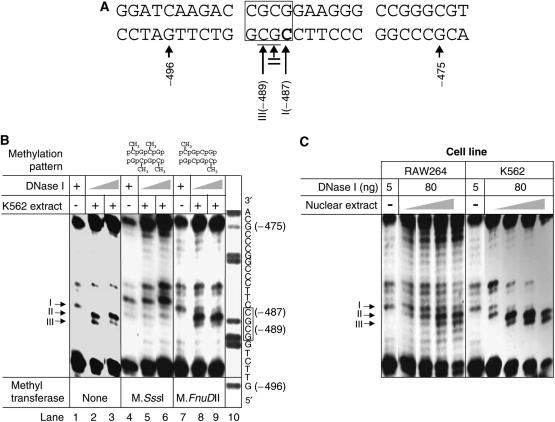
Footprint 1 site (Fp1). (**A**) Sequence of the footprint 1 site. The ciphers I, II and III correspond to the footprinting positions from the DNase I protection assay. The DNase I-protected position is shown in bold and DNase I-hypersensitive positions are underlined. The recognition sequence of M.*FnuD*II is boxed. (**B**) DNase I protection assays. Methylation of the promoter fragment modifies binding of nuclear extract proteins to footprint 1 site. Compared to the promoter fragments partially digested by DNase I alone (lane 1), addition of nuclear extracts (90 *μ*g) from K562 cells changed the digestion pattern. While one signal (I) is reduced, the intensity of two other bands is increased (II and III). M.*Sss*I methylates every cytosine residue in a CpG dinucleotide, and this methylation abolishes binding of *trans*-acting factors (lanes 4–6 are essentially identical). M.*FnuD*II only methylates the lateral cytosin residues in a CpG tetranucleotide and apparently this hemimethylation does not inhibit protective binding of *trans*-acting factors (lanes 7–9 are essentially identical to lanes 1–3). The A+G ladder (lane 10) shows the reverse complementary sequence. Numbers refer to positions relative to A in the START-ATG (=+1); the M.*FnuD*II recognition sequence is boxed. (**C**) DNase I protection assays: The binding of the *trans*-acting factors is not cell specific. DNase I protection assay with increasing amounts (20, 50, 90 and 120 *μ*g) of nuclear extracts. Band I loses intensity as the amount of nuclear extracts is increased, whereas bands II and III gain intensity. Thus, band I is protected by protein binding and bands II and III are hypersensitive to DNase digestion by protein binding, regardless of the source of the nuclear extract.

*In vitro* DNase I protection assays revealed a putative protein-binding site from position −475 to −496 (Fp1; Figure 2), which was apparent from the changes in the DNase I restriction pattern on adding nuclear extract (Figure 2B, lane 1 *vs* lanes 2+3). This protein DNA interaction proved to be methylation sensitive, as methylating the DNA by using M.*Sss*I abolished the interaction (Figure 2B, lane 4 *vs* lanes 5+6). However, hemimethylating the DNA with M.*FnuD*II failed to prevent protein loading to the DNA (Figure 2B, lane 7 *vs* lanes 8+9).

Next, we checked whether protein loading to the *GRAF* promoter is cell type specific. However, we did not detect any difference in the DNase I restriction pattern when performing protection assays in the presence of nuclear extracts prepared from either K562 or RAW264 cells, a mouse macrophage cell line (Figure 2C).

**Figure 3:**
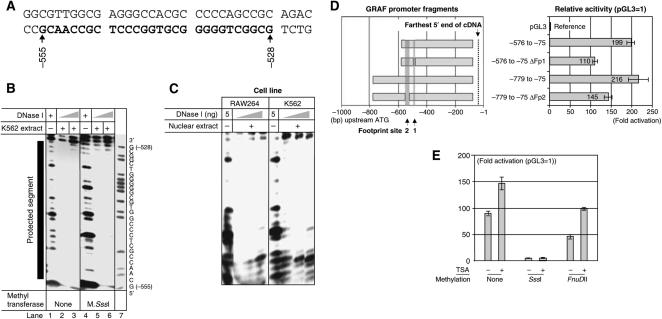
Footprint 2 site (Fp2). (**A**) Sequence of the footprint 2 site (Fp2). The sequence marked with bold is protected from DNase I digestion by binding of *trans-*acting factors. (**B**) DNase I protection assays: The segment −555 to −528 is protected from DNase I digestion (lanes 2 and 5: 40 ng; lanes 3 and 6: 80 ng DNase I) by binding of *trans*-acting factors from nuclear extract from K562 cells (90 *μ*g). This binding is insensitive to M.*Sss*I methylation of the DNA, as the DNA methylation does not alter the restriction pattern. The protected segment is marked by the black bar. The A+G ladder (lane 7) shows the reverse complementary sequence. Numbers refer to positions relative to A in the START-ATG (=+1). (**C**) The interaction of proteins with the Fp2 region of the *GRAF* promoter is not cell specific. Nuclear extracts from both RAW264 and K562 cells (90 *μ*g) can protect unmethylated DNA from digestion with increasing amounts of DNase I (40, 80 and 180 *μ*g). (**D**) Deletion of the footprint sites Fp1 and Fp2 reduces the promoter activity. Left panel: The promoter segments (numbering relative to A (=+1) in the START-ATG codon). Right panel: Relative luciferase activity normalised against the promoting activity of the empty pGL3 plasmid (=1). The data represent the mean±s.d. of three independent experiments. (**E**) Promoting repression by the M.*FnuD*II hemimethylation is partially reversible by incubation with the histone deacetylase inhibitor TSA, whereas CpG methylation with M.*Sss*I causes a complete block of the promoting activity, which cannot be restored by incubation with TSA. The reporter activities are shown as folds of increase of luciferase activity compared to the empty pGL3 plasmid. The data represent the mean±s.d. of three independent experiments.

A second putative binding site, footprint site (Fp2), was identified (Figure 3). For Fp2 however, protein binding was not methylation sensitive (Figure 3B) and, as for Fp1, the protein loading was not cell type specific (Figure 3C).

Confirmatory deletions of the two putative protein-binding sites from the promoter reduced the promoting activities in dual luciferase reporter assays (Figure 3D). Dual luciferase reporter assays with unmethylated and M.*FnuD*II- or M.*Sss*I-methylated promoter constructs demonstrated methylation sensitivity of the promoter. Thus, pGL3 constructs containing an unmethylated *GRAF* promoter showed higher relative activity than M.*FnuD*II-methylated constructs, which had a higher relative activity than the completely methylated M.*Sss*I constructs. The relative activity of the M.*FnuD*II-methylated constructs could be partially restored by incubating the cells with an inhibitor of histone deacetylase, TSA. However, TSA could not restore the activity of the fragments completely methylated by M.*Sss*I (Figure 3E).

### Influence of chromatin modifications on *GRAF* expression

**Figure 4:**
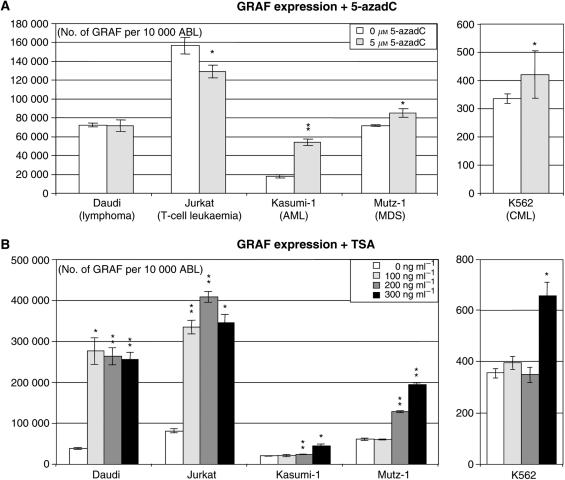
*GRAF* expression levels in cell lines after treatment with 5-azadC and TSA. (**A**) Expression levels of *GRAF* analysed by quantitative RT–PCR relative to a housekeeping gene (*ABL*) in cells treated with 5 *μ*M 5-azadC for 72 h *vs* untreated controls. ^*^P<0.05; ^**^P<0.005 (*t*-test of untreated *vs* treated). The data represent the mean±s.d. of three independent experiments. (**B**) Expression levels of *GRAF* by quantitative RT–PCR relative to a housekeeping gene (*ABL*) in cells treated with 0–300 ng TSA per ml culture medium for 16 h. ^*^P<0.05; ^**^P<0.005 (*t*-test of untreated *vs* treated). The data represent the mean±s.d. of three independent experiments.

To test the influence of a demethylating agent on the *GRAF* expression in five leukaemia and lymphoma cell lines, they were treated with 5-azadC during at least one replication cycle. 5-Aza-2′-deoxycytidine is a nucleotide analogue inhibiting DNA methyltransferase 1, which has to be incorporated into newly synthesised DNA. An integration rate of 0.3% is sufficient to inhibit 95% of methyltransferases (Creusot *et al*, 1982), as the enzymes become covalently bound to 5-azadC. The subsequent demethylation of the DNA leads to an activation of genes previously inactivated by methylation (Juttermann *et al*, 1994). Quantitative PCR analyses of 5-azadC-treated cells *vs* untreated cells revealed an increase of *GRAF* expression in Mutz-1 and K562 and a highly significant increase in the AML cell line Kasumi-1 (Figure 4A). Morphologically, we did not notice any changes (data not shown).

To test the influence of the histone deacetylase inhibitor TSA, five leukaemia and lymphoma cell lines were treated with 100–300 ng TSA per ml culture medium for 16 h. Quantitative PCR showed an enhanced *GRAF* expression in all cell lines examined, but the TSA concentration necessary to achieve this effect varied. In the myeloid cell line Mutz-1, 100 ng ml^−1^ TSA had no effect, whereas 200 and 300 ng ml^−1^ TSA elevated the *GRAF* expression level two- and three-fold *vs* the untreated controls. In Kasumi-1 and K562, 300 ng ml^−1^ was necessary to double the *GRAF* expression. In contrast, treating the Jurkat (derived from T-cell leukaemia lymphoma) and Daudi (lymphoma) cell lines with 100 ng ml^−1^ TSA elevated the *GRAF* expression four- and seven-fold compared to the untreated controls (Figure 4B). The influence of a combination of TSA and 5-azadC on *GRAF* expression levels has not been tested.

### CpG methylation of the *GRAF* promoter in patients with AML/MDS

**Figure 5:**
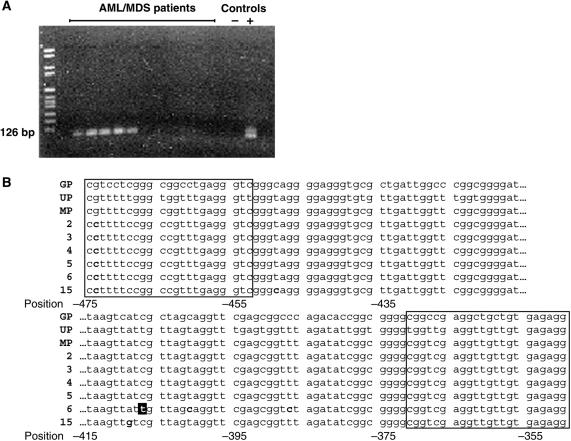
Methylation-sensitive PCR of the *GRAF* promoter in patients’ samples. (**A**) Methylation-sensitive PCR. A 126 bp fragment of the *GRAF* promoter (segment −475 to −350) was amplified by applying MS-PCR. An artificially M.*Sss*I-methylated promoter construct served as a positive control. (**B**) Sequences of MS-PCR products. Alignment of six PCR products (−475 to −350) generated from patient samples showing complete methylation of CpG dinucleotides within the *GRAF* promoter. Numbering refers to patients in Table 2. GP=germline promoter sequence; UP=unmethylated promoter sequence after MS-PCR; MP=methylated promoter after MS-PCR. Bold cytosine and guanine residues indicate sequence variations and inverted thymidine residues indicate a lack of methylation. Primer sequences are framed.

Methylation-sensitive PCR was used to reveal the presence of a deactivating CpG methylation in the 5′ flanking region. Bone marrow and peripheral blood from 37 healthy donors and bone marrow samples from 29 patients with AML or MDS were analysed by MS-PCR and sequencing of the MS-PCR-positive products. Among the healthy individuals, only one out of 37 showed a positive MS-PCR (2.7%). The sequence of this PCR product revealed methylation of the cytosine residues at the primer-binding sites but not within the PCR product. We therefore interpret this as a technical error. In 11 out of 29 patient samples (38%), a PCR product was generated, indicating a methylated *GRAF* promoter (Figure 5A and Table 2). Six PCR products were sequenced showing a complete methylation of the cytosine residue in CpG dinucleotides (Figure 5B). Unfortunately, lack of sufficient patient material prevented us from testing the GRAF protein expression by immunohistochemical analysis or to correlate *GRAF* methylation status with *GRAF* expression.

## DISCUSSION

The *GRAF* gene located at chromosome 5q31 is a rare fusion partner of *MLL* in cases of AML. So far, the fusion has been reported in only three cases, all resulting in similar clinical features (Borkhardt *et al*, 2000; Panagopoulos *et al*, 2004; Wilda *et al*, 2005). In the present study, we identify and characterise the regulative region of the *GRAF* gene. The *GRAF* promoter lacks a TATA or CCAAT box, but the transcription initiation site is flanked by a CpG island as described for other genes (Gardiner-Garden and Frommer, 1987).

CpG islands are well-known targets of epigenetic modifications. Methylation of cytosine residues is responsible for silencing of transposable elements, for defence against viral sequences and for the transcriptional repression of genes. The methylation status is heritable through cell divisions and maintained by a number of DNA methyltransferases (Egger *et al*, 2004).

To address the question whether *GRAF* expression might be compromised by other mechanisms in patients without *MLL-GRAF* fusion, we examined bone marrow samples from patients with AML or MDS. In previous studies, three out of 13 samples with loss of one 5q allele exhibited a disruption of the second *GRAF* allele by insertions (Borkhardt *et al*, 2000). Therefore, if *GRAF* has tumour-suppressing abilities, we assume that additional mutations or epigenetic modifications contribute to the malignant phenotype by downregulation or complete abolishment of *GRAF* expression. The involvement of epigenetic modifications such as promoter methylation is well known for a variety of established tumour suppressor genes, the most prominent examples being the retinoblastoma gene product and the cyclin-dependent kinase inhibitor CDKN2A. For both genes, dense hypermethylation of the promoter leads to a loss of gene expression (Herman, 1999). Likewise, epigenetic events (hypermethylation) in the genes *hMLH1, p16INK4A* and *GSTPI* can also lead indirectly to tumour progression by promoting genetic instability in several tumour entities, such as breast, prostate, gastric or colon cancer (Baylin and Herman, 2000; Herman and Baylin, 2003). Along these lines we show a repression of activity owing to methylation of the *GRAF* promoter. Two areas with special importance for the activity of the promoter have been identified and examined by DNase I protection assays (footprint sites Fp1 and Fp2). These *in vitro* assays indicated the binding of two factors to the *GRAF* promoter. Presumably, both factors are not expressed in a cell-specific manner. The Fp1 region contains three CpG dinucleotides of which an incomplete methylation by M.*FnuD*II did not interfere with the binding of the protein factor. Only complete methylation by M.*Sss*I abolished the binding, indicating an interference of the methylated 3′ cytosine within the methylase recognition site (CGCG) with the DNA binding of the protein. Thus, the interaction of the protein with Fp1 is methylation sensitive, as has been found for a variety of transcription factors such as CREB, NF-*κ*B, E2F or AP2 (Kovesdi *et al*, 1987; Iguchi-Ariga and Schaffner, 1989; Comb and Goodman, 1990; Bednarik *et al*, 1991; Baylin and Herman, 2000; Herman and Baylin, 2003).

A second factor interacts with footprint site 2 (Fp2) but without exhibiting methylation sensitivity. Similar behaviour has been demonstrated for the transcription factors CTF and YY1 (Ben Hattar *et al*, 1989; Gaston and Fried, 1995). Nevertheless, it is possible that modifications of the chromatin structure as a consequence of the DNA methylation block the binding of a protein factor *in vivo* although the factor is methylation insensitive *in vitro*, as is known for NF-Y (Ammerpohl *et al*, 1997, 1998). As our attempt to identify both binding factors by yeast one-hybrid assays failed, these important transcriptional regulators have to be identified in further studies.

Methylation of the *GRAF* promoter in patients with AML or MDS was examined by MS-PCR, in which methylated and unmethylated alleles are distinguished by conversion of all unmethylated, but not of the methylated, cytosine to uracil residues with bisulphite (Herman *et al*, 1996). In 38% of AML and MDS patients, we detected methylation of the *GRAF* promoter. Within a control group of 37 blood donors, only one incompletely methylated *GRAF* promoter was detectable (3%), indicating a highly increased rate of *GRAF* promoter methylation in haematologic disorders of myeloid origin.

*GRAF* expression is also influenced by the histone acetylation status. Acetylation and deacetylation of nucleosomal histones are important cellular tools for the regulation of gene expression. Hyperacetylated histones are associated with gene activity whereas a lack of acetylation leads to the repression of gene expression (Marks *et al*, 2003). These epigenetic modifications are catalysed by two groups of enzymes, histone acetyl transferases and histone deacetylases (HDACs). Inhibition of HDACs is thought to augment gene transcription, as the histones cannot be changed to their highly condensed, hypoacetylated form (Kim *et al*, 2003). Application of TSA leads to an enhancement of *GRAF* expression, especially in the non-myeloid cell lines tested. The effect was dose dependent in the myeloid cell lines, which required at least twice as much TSA as the non-myeloid cell lines, indicating an additional layer of modification regulating *GRAF* expression in these cells. Trichostatin A induces significant changes in gene expression in only 2% of all genes (Marks *et al*, 2001), so histone acetylation seems to play a specific role in the regulation of *GRAF* gene expression. The different developmental lines in haematological differentiation therefore seem to become manifest in different *GRAF* chromatin patterns. Histone deacetylase inhibitors are tested in clinical trials, as they are known to induce growth arrest, differentiation and/or apoptosis in cells of various tumour types (Marks *et al*, 2000, 2001).

It has been shown extensively by others that treatment of leukaemia cell lines employed in our work (Daudi, Jurkat, Kasumi-1, Mutz-1 and K562) with the HDAC inhibitor TSA or the DNA methyltransferase 1 inhibitor 5-azadC has a profound impact on the expression of several genes and decreases proliferation and clonogenic survival and increases the level of apoptotic markers (Blagosklonny *et al*, 2002; Zhu and Otterson, 2003; Karagiannis *et al*, 2004; Liu *et al*, 2004; Yokota *et al*, 2004; Furukawa *et al*, 2005). Therefore, it is not surprising that the expression level of *GRAF* seems to be influenced as well. Our findings are consistent with the fact that treatment of cells with 5-azadC leads to an enhancement of *GRAF* expression only in myeloid cells, especially in a cell line derived from an AML. However, additional work is necessary to characterise the nature of these interactions in more detail in myeloid leukaemia *vs* MDS. Whether CpG methylation of the *GRAF* promoter and/or the *GRAF* expression level could improve the initial diagnosis, stratification or prognostication of patients with myeloproliferative diseases should be addressed in future studies including a large number of patients.

In conclusion, varying mechanisms (translocations, allelic loss, insertions, promoter methylation and probably chromatin modification) leading to an inactivation of *GRAF* can be observed in a subset of AML and MDS cases. The pathogenetic role of aberrant *GRAF* expression in these diseases remains to be established.

## Figures and Tables

**Table 1:** Primer list

**Assay**	**Orientation**	**Nucleotide sequence (5′ → 3′)**
*Rapid amplification of cloned ends*		
1	Adaptor primer 1, forward	CCATCCTAATACGACTCACTATAGGGC
1	Gene-specific primer 1, reverse	AATTTGTTGGTCTTGTCCAGCTCTGCTTCGTGCG
2	Adaptor primer 2, forward	ACTCACTATAGGGCTCGAGCGGC
2	Gene-specific primer 2, reverse	GCACCGCGGCTTGAGCGTCTCTCGGAAGTGCGGACTATCGAG
		
*Methylation-sensitive PCR*		
1	Forward	GATTAAGATCGCGGAAGGGTCG
1	Reverse	AACTCTCGACAACCTCGAACGC
2	Forward	CGTTTTCGGGCGGTTTGAGGGTC
2	Reverse	CCTCTCACAACAACCTCGACCG
		
*Real-time PCR*		
1	GRAF-F	CAGAACATTGTCATTGAGATCCTAATAGA
1	GRAF-R	GGGCATTGGTGAGAGGCATA
1	GRAF probe	AACCACGAAAAGATATTTAACACCGTGCCCGA
1	ABL-F	CAACACTGCTTCTGATGGCAA
1	ABL-R	CGGCCACCGTTGAATGAT
1	ABL probe	CAACACCCTGGCCGAGTTGGTTCAT

**Table 2:** Results of the MS-PCR

**Sample**	**Diagnosis, subtype**	**MS-PCR result**
1	AML, M7	−
2	AML, not classified	+
3	AML, M5	+
4	AML, M5	+
5	AML, M5	+
6	AML, not classified	+
7	AML, M5	−
8	AML, M4	−
9	AML, M4	−
10	AML, M2	−
11	AML, M2	−
12	AML, M2	−
13	AML, M2	−
14	AML, M2	−
15	AML, M2	+
16	MDS, JMML	+
17	MDS, RAEB-T, ANLL	+
18	MDS, RA	−
19	MDS, RA	−
20	MDS, CMML	−
21	MDS, CMML	+
22	MDS, not classified	−
23	MDS, CMML	+
24	MDS, not classified	+
25	MDS, RAEB	−
26	MDS, RA	−
27	MDS, RA	−
28	MDS, RAEB	−
29	MDS, not classified	−

MS-PCR=methylation-sensitive PCR; AML=acute myeloid leukaemia; MDS=myelodysplastic syndrome.

## Supplementary Material

Supplementary Date
