# Supplementary material for: Characterisation of the GRAF gene promoter and its methylation in patients with acute myeloid leukaemia and myelodysplastic syndrome
Source: Br J Cancer. 2006 Jan 10;94(2):323–32. doi: 10.1038/sj.bjc.6602939 (PMC2361128; doi:10.1038/sj.bjc.6602939)
Supplement: Supplementary Date [file 94-6602939x1.pdf]

Supplementary data

With the primers noted in Table A and 1ng of a cloned putative *GRAF*-promoter as the template, it was possible to generate different fragments of the 5'-flanking region of *GRAF*. After purification (QIAquick Spin Kits from Qiagen, Hilden, Germany) and restriction (*Hind*III and *Bgl*II, Boehringer Mannheim, Germany) using conventional methods the PCR-products were cloned into the *Hind*III/*Bgl*II digested pGL3-BasicVector (Promega, Mannheim, Germany).

Table A

Primer sequences for the generation of promoter fragments

Artificial restriction sites: Sense primers: AGATCT, *Bgl*II, #

Antisense primers: AAGCTT, *Hind*III, §

**Name**	**First base**	**Nucleotide sequence (5'→3')**	**Last base**	**Orientation**
Prom up 1482	-1482	§+#CACGTAGCTGAAGGAGGGCTGCTGGTTCC	-1454	Sense
Prom up 981	-981	§+#ACCGTGGGCGGGTTCTGAGCAGGAGTGGCC	-952	Sense
Prom up 576	-576	#GAGTTTGCGCTGCGGACCTAGGCGTTGG	-560	Sense
Prom up 507	-507	#CTTTGATTGGATCAAGACCGCGGAAGGGCC	-478	Sense
Prom up 242	-242	#GTTTCCTGCTCGCGATCCGCTCCGTTGCC	-214	Sense
Prom do 341	-341	§TCGAGCGCCCTCTCACAGCAGCCTCGGCC	-369	Antisense
Prom do 176	-176	§GAGCTCGGACCCGGCCGAGGTGCTGCTGTTCC	-206	Antisense
Prom do 158	-158	§CGCAAGACTCCCGAAGCGAGCTCGGACC	-185	Antisense
Prom do 108	-108	§GGCTCCACAGGTGTGGCGCTGGCTCACTCC	-137	Antisense
Prom do 75	-75	§GCGGGACCCCGGCCGGCTCCCCCGACGGCC	-104	Antisense
Prom do 52	-52	§CCGCCGCCCAGAGCACTCACGCGGCGGGAC	-81	Antisense
Prom do	-1	§+#GTGCGCGCGGCGCCCATCCGGGGGGCGCGCC	-31	Antisense

Table B

Mutagenesis-primer for promoter constructs with deletion of the footprint regions

Name	First base	Nucleotide sequence (5'→3')	Last base	Orientation
Prom s Fp1	-483	GGGCCGGGCGTCCTCGGGCG	-464	Sense
Prom a Fp1	-494	TTGATCCAATCAAAGGGGCC	-513	Antisense
Prom s Fp2	-523	CCTGCCTCCAGGCCCCTTTG	-504	Sense
Prom a Fp2	-554	CGCCTAGGTCCGCAGCGCAAAC	-575	Antisense

Table C

Buffers used in the Preparation of nuclear extracts and DNase I protection assays

Buffer name	Composition
Hypotonic buffer	10mM Hepes; 0.75mM spermidine; 0.15mM spermine; 0.1mM EDTA; 0.1mM EGTA; 1mM DTT; 10mM potassium chloride; 10mM benzamidine; 0.5mM PMSF
Restore buffer	68% sucrose; 50mM Hepes; 0.75mM spermidine; 0.15mM spermine; 10mM potassium chloride; 0.2mM EDTA; 1mM DTT
Resuspension buffer	20mM Hepes; 0.75mM spermidine; 0.15mM spermine; 0.2mM EDTA; 0.2mM EGTA; 2mM DTT; 25% glycerol; 10mM benzamidine; 0.5mM PMSF; mix 9 volumes of this buffer with 1 volume ammonium sulfate solution (4M)
Buffer D	20mM Hepes; 20% glycerol; 0.1M potassium chloride; 0.2mM EDTA; 0.5mM PMSF; 0.5mM DTT
Binding buffer	10mM Tris-HCl; 1mM EDTA; 0.1mM EGTA; 1mM DTT; 60mM potassium chloride; 5% glycerol
Stop buffer	0.5M sodium acetate; 30mM EDTA; 1%SDS; 0.2μg/ml glycogen

Figure legends

Supplementary Figure 1 Deletion analyses (**B**) of the *GRAF* promoter.

Left panel: The promoter segments (numbering relative to A (=+1) in the START-ATG codon). Right panel: Relative Firefly luciferase activity normalized against the promoting activity of SV40 (=100%). The data represent the mean ± SD of three independent experiments.
