# Supplementary figures and images for: Characterisation of the GRAF gene promoter and its methylation in patients with acute myeloid leukaemia and myelodysplastic syndrome
Source: Br J Cancer. 2006 Jan 10;94(2):323–32. doi: 10.1038/sj.bjc.6602939 (PMC2361128; doi:10.1038/sj.bjc.6602939)

**GRAF Promoter fragments**

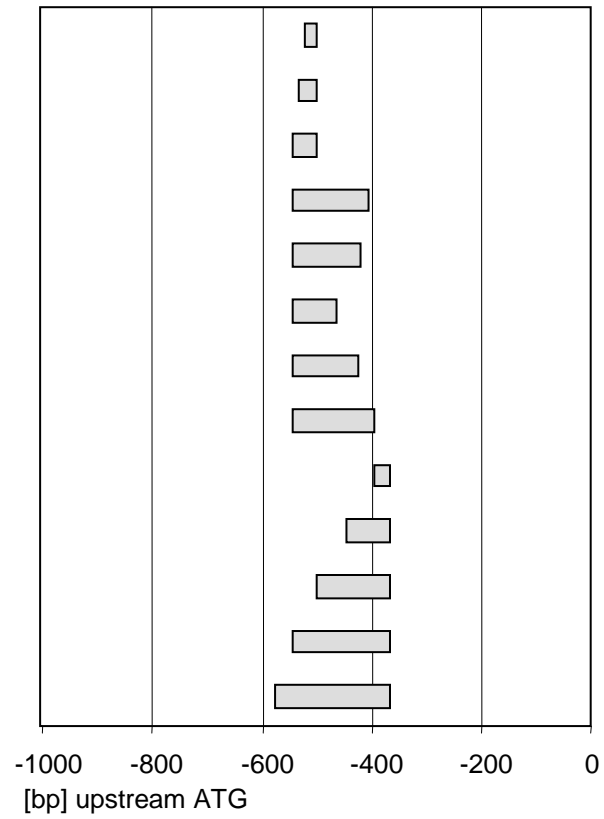

**Relative activity (SV40= 100%)**

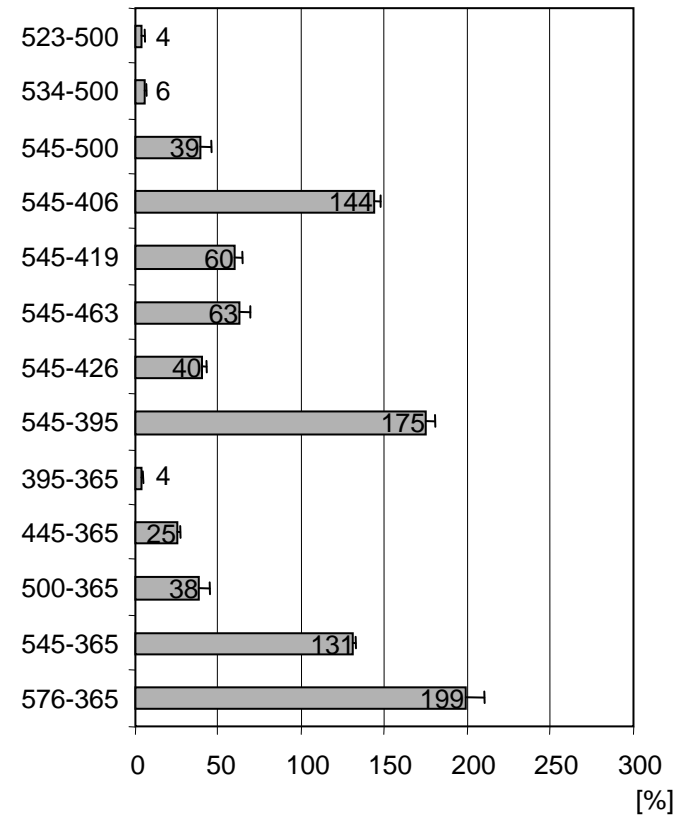

Supplement: Supplementary Figure [file 94-6602939x2.pdf]
